# Fear of progression among postoperative patients with newly diagnosed lung cancer: a cross-sectional survey in China

**DOI:** 10.1186/s40359-023-01211-5

**Published:** 2023-05-22

**Authors:** Ruiyun Chen, Hui Yang, Hongmei Zhang, Jingru Chen, Saisai Liu, Li Wei

**Affiliations:** 1 Department of Thoracic Surgery, Henan Key Laboratory for Nursing, Henan Provincial People’s Hospital, People’s Hospital of Zhengzhou University, Henan University People’s Hospital, No.7 Weiwu Road, Jinshui District, Zhengzhou, 450003 Henan People’s Republic of China; 2 Department of Nursing, Henan Key Laboratory for Nursing, Henan Provincial People’s Hospital, People’s Hospital of Zhengzhou University, Henan University People’s Hospital, Zhengzhou, 450003 Henan People’s Republic of China

**Keywords:** Lung cancer, Fear of progression, Newly diagnosed, Related factors

## Abstract

**Background:**

More lung cancer cases are becoming diagnosed earlier in recent years. The diagnosis is often accompanied by fear of progression (FoP). There is a clear research gap in the existing literature on FoP and the most frequent concerns in newly diagnosed lung cancer patients.

**Objective:**

To identify the status and factors related to FoP in newly diagnosed Chinese lung cancer patients undergoing thoracoscopic lung cancer resection.

**Methods:**

A cross-sectional design with convenience sampling was used in this study. Participants (N = 188) with newly diagnosed lung cancer (≤ 6 months) at one hospital in Zhengzhou were recruited. A demographic questionnaire, Fear of Progression Questionnaire-Short Form, Social Support Rating Scale (SSRS), Simplified Coping Style Questionnaire, and Brief Illness Perception Questionnaire were used to assess characteristics, FoP, social support, coping style, and patient illness perceptions. Multivariable logistic regression analysis was used to identify factors associated with FoP.

**Results:**

The mean score of FoP was 35.39 ± 8.03. There are 56.4% of the patients (scores ≥ 34) have a clinically dysfunctional level of FoP. FoP was higher in young (18–39 years) than middle-aged patients (40–59 years) and elderly patients (≧60 years) (*P* = 0.004). Patients aged 40–59 years showed significantly higher fear of family-related concerns (*P* < 0.001), a fear of potential harm from medications (*P* = 0.001); Patients aged 18–39 years and 40–59 years showed significantly higher fears of work-related concerns (*P* = 0.012). Multiple logistic regression analyses showed that patients’ age, the time from surgery and SSRS score were found to be independently associated with higher FoP.

**Conclusions:**

High FoP is a frequently reported problem among newly diagnosed lung cancer patients, especially those less than 60 years old. Professional psychoeducation, psychological interventions, and personalized support are needed for patients with a high FoP.

## Introduction

Cancer is undoubtedly one of the most critical global public health concerns and is the leading cause of disease-related death. Likewise, lung cancer is one of the most common malignant tumors. According to the 2020 Global Cancer Statistics Report [1], there were 19.29 million new global cancer cases, including 4.57 million new cancer cases in China, accounting for 23.7% of the global total. Furthermore, 820,000 of those cases in China were related to lung cancer. As China is the world's most populous country, the incidence and mortality of lung cancer are 11.4% and 18%, respectively, which ranks first in the incidence and mortality of malignant tumors in China [1].

More lung cancer cases are becoming diagnosed earlier in recent years with the improvement of people's awareness of medical examination and the popularization of early screening technology for high-risk groups. Consequently, the survival rate of postoperative patients with lung cancer has also significantly increased due to these advancements and efforts. Even so, a lung cancer diagnosis is often accompanied by worries and fears. Generally, fear of cancer recurrence (FCR) refers to the psychological state of patients who are afraid that the disease may recur or progress [2]. It is also described as fear of progression (FoP), or concern about cancer relapse or return [3, 4]. However, this definition conflates FCR and FoP, which is virtually comparable at a conceptual level, and there is indeed very strong overlap between them for practical purpose [5]. Conceptually, FCR seems most relevant to those who have entered remission but fear their cancer returning [6]. FoP describes an adequate and realistic response to aextraordinary life events, such as the threatening diagnosis of cancer [7]. The latest research tests Fardell et al.'s cognitive processing model [8], it clearly shows that FCR and FoP are not the same construct, nor are they associated with the same psychological variables. The factor analysis demonstrates that items from the Fear of Cancer Recurrence Inventory (FCR-I) severity subscale and Fear of Progression Questionnaire-Short Form (FoP-Q-SF) loaded on separate, but related, factors. The structural modelling shows that FCR and FoP are related with some overlapping predictors, such as metacognitions, intrusions and perceived risk of recurrence, other differences emerged. Risk perception and bodily threat monitoring are more strongly associated with FCR than FoP. However, both FCR and FoP are associated with metacognitions and intrusions [6]. Therefore, the findings have important clinical implications, it is essential to distinguish between FoP and FCR with specific instruments and providing optimal care, particularly to the increasing number of survivors living with advanced disease.

FoP is considered as an appropriate, rational response to the real threat of cancer and related treatments [9]. It is one of the most common psychological burdens and unmet needs that many cancer survivors experience and is challenging to ignore [10]. It has demonstrated that most patients reported low or moderate level of FoP [7], and still suffer from it even years after their initial diagnosis [11]. It is reported that around 36% of Chinese adolescent and young adult patients experience a dysfunctional level of FoP [12]. Additionally, added research has reported that the prevalence rates of FoP experienced by lung cancer survivors range between 37 and 77.93% [13]. However, patients with severe FoP may suffer from sleep disturbances, depression and poor quality of life, and may require added health care services [14]. Thus, the assessment of FoP in lung cancer survivors is necessary for improving their management and updating effective interventions to alleviate FoP in patients.

Currently, the theoretical models and conceptual framework related to FoP are drawn from cognitive behavioral paradigms, including the self-regulation model of illness (SRMI) [15], self-regulatory executive functioning model [16], social-cognitive processing model (SCPM) [17, 18], and the uncertainty in illness theory [19]. SRMI assumes that the key factor of FoP is illness perception (IP), emphasizing that individuals react negatively to trigger factors due to their negative cognition of disease, thus guiding them to make different coping styles [8]. The SCPM model suggests that social networks play an important role in promoting the cognitive processing of cancer-related memories, thoughts, and concerns, which may enhance phychological adjustment [8]. Previous investigations have suggested that a variety of factors associated with FoP in adult cancer survivors included younger age, gender, marital status, residence, employment, duration since initial diagnosis, and stage of the disease [20]. Likewise, several other associated factors such as social support, perception of the illness, psychological distress, and quality of life have been suggested to act as predictors of FoP [21–23]. For example, Wang [21] found that IP was not only positively correlated with FoP but was also an independent predictor of FoP for lung cancer patients. However, the associations among these variables in newly diagnosed lung cancer patients remain to be elucidated.

Newly diagnosed cancer patients will suffer from worry, overwhelming fear, and depression from the time of initial diagnosis. However, much of the evidence is inconclusive when investigating the association between the duration of initial diagnosis and FoP [20]. Up to date, only a few studies on FoP were conducted in cancer patients [24–26]. One investigation showed that 16.9% of the elderly breast cancer patients experiencing high and 66.2% experiencing moderate levels of FoP ten weeks after the surgery [24]. Another study found that nearly 65% of the newly diagnosed cancer patients reported high FoP [25]. Additionally, there is little evidence concerning the psychology of newly diagnosed lung cancer patients. Considering the unique characteristics of this population, we should make more efforts to understand and provide better insights into them. However, previous studies on FoP have focused on different populations of cancer patients, and the duration since initial diagnosis (as in time to confirm diagnosis) was typically more than 6 months. Therefore, there is a clear research gap in the existing literature on FoP and the most frequent concerns in newly diagnosed lung cancer patients. Given these situations, our study focused on newly diagnosed Chinese lung cancer patients with a duration of diagnosis ≤ 6 months. This study aims to identify factors associated with FoP in lung cancer patients, depict the difference between young (18–39 years), middle-aged (40–59 years) and elderly patients (≧60 years) with FoP, and explore the relationships of FoP with social support, coping style, and illness perception.

## Methods

### Study design

The cross-sectional study was conducted in China from January 2021 to September 2021. We recruited patients from the thoracic surgery department in Henan Provincial People’s Hospital.

### Participants

Patients were eligible for study participation if they had met the inclusion criteria: (1) age ≥ 18 years at the time of diagnosis, (2) had a new diagnosis of lung cancer within 6 months, (3) had a thoracoscope lung resection surgery; (4) able to speak and write Chinese; (5) provide written informed consent according to the Declaration of Helsinki. Participants were excluded if they: (1) had severe physical, verbal and/or cognitive impairment; (2) transferred departments due to changes in condition; (3) had other tumors or received adjuvant therapy such as radiotherapy and chemotherapy before operation; (4) had any comorbid psychiatric conditions. G*Power version 3.1 software was used to identify the sample size. With 0.9 power and 15 demographic and research variables, 171 patients were needed to participate in the study [27].

### Recruitment and data collection

All potential participants who met all eligibility criteria were invited to participate in the study. They were approached in the chat room by trained research nurses who explained the study's aim, how to complete the questionnaire and its confidentiality. Those who agreed to participate were asked to read a personal information sheet and then requested to sign the written informed consent. Then the participant was given an online questionnaire with face-to-face instructions. For those without higher education levels and unable to read the questionnaire, the research nurses explained the items one by one and assisted them in filling out the form.

### Measures

#### Demographic and clinical characteristics

Basic demographic and clinical characteristics were self-reported by participants. The detailed sheet of collected data included: gender, age, marital status, education level, residence, employment, personal monthly income, religious belief, chronic diseases, stage of disease at initial diagnosis, the time from surgery and Chemotherapy/radiotherapy after surgery (Yes or No).

#### Fear of Progression Questionnaire-Short Form, FoP-Q-SF

The 12-item short version of the Fear of Progression Questionnaire-Short Form (FoP-Q-SF) was used to measure patient FoP in this study. The scale was developed in 2006 [28] and was adapted in Chinese in 2016 [29]. It consists of 12 items, including two dimensions of physical and social/family well-being, which have been successfully applied to patients with chronic diseases such as cancer. The items are scored on a 5‐point Likert Scale ranging from 1 to 5 (never to very often), with a total score ranging from 12 to 60. A higher score indicates a higher FoP for the patient, and scores ≥ 34 indicate a clinically dysfunctional level of FoP [7]. The psychometric properties are validated and reliable, and Cronbach’s alpha is 0.886 [29].

#### Social Support Rating Scale, SSRS

The SSRS assessed the patient’s social support. It was developed in 1986 [30], and is a 10-item self-reported measure that includes three dimensions of objective, subjective, and availability support. It is commonly used to understand the social support of different individuals. Its total scores range from 12 to 66, and a higher score indicates a higher level of social support that the individual feels. The Chinese version of SSRS shows good psychometric properties, and Cronbach’s alpha is 0.856.

#### Simplified Coping Style Questionnaire, SCSQ

The SCSQ was developed in 1978, revised, and simplified in Chinese in 1998 [31]. It is a self-report measure used to evaluate individuals' coping styles. It is comprised of 20 items, including two dimensions of active coping (twelve items, e.g., “be relieved through work, study or some other activities.”) and negative coping (eight items, e.g., “try to forget the whole thing.”), which refers to the cognitive and behavioral patterns adopted by individuals when they encounter setbacks and pressures. The items are scored on a 4‐point Likert Scale ranging from 0 to 3 (not taking a coping style to often taking a coping style), with a total score ranging from 0 to 60. The psychometric properties of SCSQ are validated and reliable. The Cronbach’s alpha is 0.9, and the retest correlation coefficient is 0.89 [31].

#### Brief Illness Perception Questionnaire, BIPQ

The BIPQ is used to measure a patient’s perception of illness [32]. The scale is a 9-item self-reported scale. The items are scored on a 10‐point Likert Scale ranging from 1 to 10 (minimum to maximum), with a total score ranging from 0 to 80. A higher score indicates a more severe perception of illness for the patient. The BIPQ has been translated and well-validated in Chinese (internal consistency = 0.91) [33]. The psychometric properties are satisfactory (Cronbach’s alpha = 0.831).

### Data analysis

All statistical analyses were performed in IBM SPSS for Windows version 26. All demographic and clinical characteristics were analyzed using descriptive statistics. Categorical variables are described as frequency and percentage, and continuous variables are described as mean and standard deviation (SD). Categorical variables (e.g., gender, marital status, stage of disease at initial diagnosis) were compared using a $\chi^{2}$ test. Continuous variables (e.g., scores of FoP, SSRS, SCSQ, and BIPQ) between different age groups Variables between the two groups (low vs. high FoP) were performed in independent sample t-test, Chi-square test, or Mann–Whitney U test, as appropriate. All significant variables (alpha level = 0.05) in the univariate analyses were further examined by a binary logistic regression multivariable model with the “enter” method to identify factors independently associated with FoP (low/high). G*Power version 3.1 software was used to calculate the power analysis, based on the effect size with a total sample of 188 patients: the power is 0.98. The two-sided Wald test was used, and *P* values < 0.05 were considered statistically significant.

## Results

### Participants' characteristics

A total of 216 survivors were screened, and 28 (12.96%) survivors were excluded due to different reasons. Overall, 188 participants were included in the final analysis (Table 1). The mean age of participants was 56.41 ± 13.12 years (range, 19–82), 23 patients were between the ages of 18–39 years with a mean age of 31.52 ± 6.19, 79 patients were between the ages of 40–59 years with a mean age of 51.53 ± 5.26, and 86 patients were ≥ 60 years with a mean age of 67.56 (SD = 5.31). The mean time from surgery was 2.97 ± 1.29 months. Of the 188 patients, approximately 99 (52.7%) participants were male, 175 (93.1%) of them were married, 119 (63.3%) of them were at Stage II of lung cancer at initial diagnosis, and 73 (38.8%) had comorbid chronic diseases (among them, 45 (23.9%) with hypertension, 15 (8.0%) with coronary heart disease, 21 (11.2%) with diabetes, 15 (8%) with cerebrovascular disease or others.)

**Table 1:** Sample characteristics

Variable	All patients (N = 188) N (%) M (SD)	18–39 years (N = 23) N (%) M (SD)	40–59 years (N = 79) N (%) M (SD)	≧60Years (N = 86) N (%) M (SD)	χ^2^/F	*P*-value
Gender					1.202	0.548
Male	99 (52.7)	11 (47.8)	39 (49.4)	49 (57.0)		
Female	89 (47.3)	12 (52.2)	40 (50.6)	37 (43.0)		
Educational level					33.326	< 0.001
Primary school or below	47 (25.0)	0 (0.0)	14 (17.7)	33 (38.4)		
Junior high school	49 (26.1)	6 (26.1)	24 (30.4)	19 (22.1)		
High school and technical secondary school	43 (22.9)	3 (13)	17 (21.5)	23 (26.7)		
College or above	49 (26.1)	14 (60.9)	24 (30.4)	11 (12.8)		
Marital status					~	0.001
Married	175 (93.1)	18 (78.3)	75 (94.9)	82 (95.3)		
Unmarried	6 (3.2)	5 (21.7)	1 (1.3)	0 (0.0)		
Separated	3 (1.6)	0 (0.0)	2 (2.5)	1 (1.2)		
Widowed	4 (2.1)	0 (0.0)	1 (1.3)	3 (3.5)		
Residence					1.497	0.473
Rural	95 (50.5)	9 (39.1)	40 (50.6)	46 (53.5)		
Town and city	93 (49.5)	14 (60.9)	39 (49.4)	40 (46.5)		
Employment					33.217	< 0.001
No	106 (56.4)	9 (39.1)	29 (36.7)	68 (79.1)		
Yes	82 (43.6)	14 (60.9)	50 (63.3)	18 (20.9)		
Family per capita income (monthly) RMB					6.461	0.374
Less than 1000	43 (22.9)	6 (26.1)	14 (17.7)	23 (26.7)		
1001–3000	64 (34)	5 (21.7)	30 (38)	29 (33.7)		
3001–5000	44 (23.4)	5 (21.7)	17 (21.5)	22 (25.6)		
More than 5000	37 (19.7)	7 (30.4)	18 (22.8)	12 (14.0)		
Religious beliefs					1.722	0.423
Yes	8 (4.3)	2 (8.7)	2 (2.5)	4 (4.7)		
No	180 (95.7)	21 (91.3)	77 (97.5)	82 (95.3)		
Chronic diseases					27.283	< 0.001
Yes	73 (38.8)	2 (8.7)	21 (26.6)	50 (58.1)		
No	115 (61.2)	21 (91.3)	58 (73.4)	36 (41.9)		
The time from surgery					14.145	0.001
0–3 months	128 (68.1)	19 (82.6)	42 (53.2)	67 (77.9)		
4–6 months	60 (31.9)	4 (17.4)	37 (46.8)	19 (22.1)		
Stage of disease at initial diagnosis					29.163	< 0.001
I	69 (36.7)	20 (87)	26 (32.9)	23 (26.7)		
II	119 (63.3)	3 (13)	53 (67.1)	63 (73.3)		
Chemotherapy/radiotherapy after surgery					7.570	0.023
Yes	56 (29.8)	2 (8.7)	30 (38)	24 (27.9)		
No	132 (70.2)	21 (91.3)	49 (62)	62 (72.1)		
SSRS score	43.79 ± 6.78	45.96 ± 6.35	44.91 ± 7.13	42.17 ± 6.25	4.892^a^	0.009
SCSQ score	52.05 ± 11.03	53.22 ± 11.46	53.05 ± 10.26	50.83 ± 11.60	0.983^a^	0.376
BIPQ score	55.74 ± 14.79	53.43 ± 16.51	56.39 ± 13.30	55.76 ± 15.70	0.354^a^	0.702

SD, standard deviation; a, *F* value; SSRS, Social Support Rating Scale; SCSQ, Simplified Coping Style Questionnaire; BIPQ, Brief Illness Perception Questionnaire

### Fear of cancer progression

**Fig. 1:**
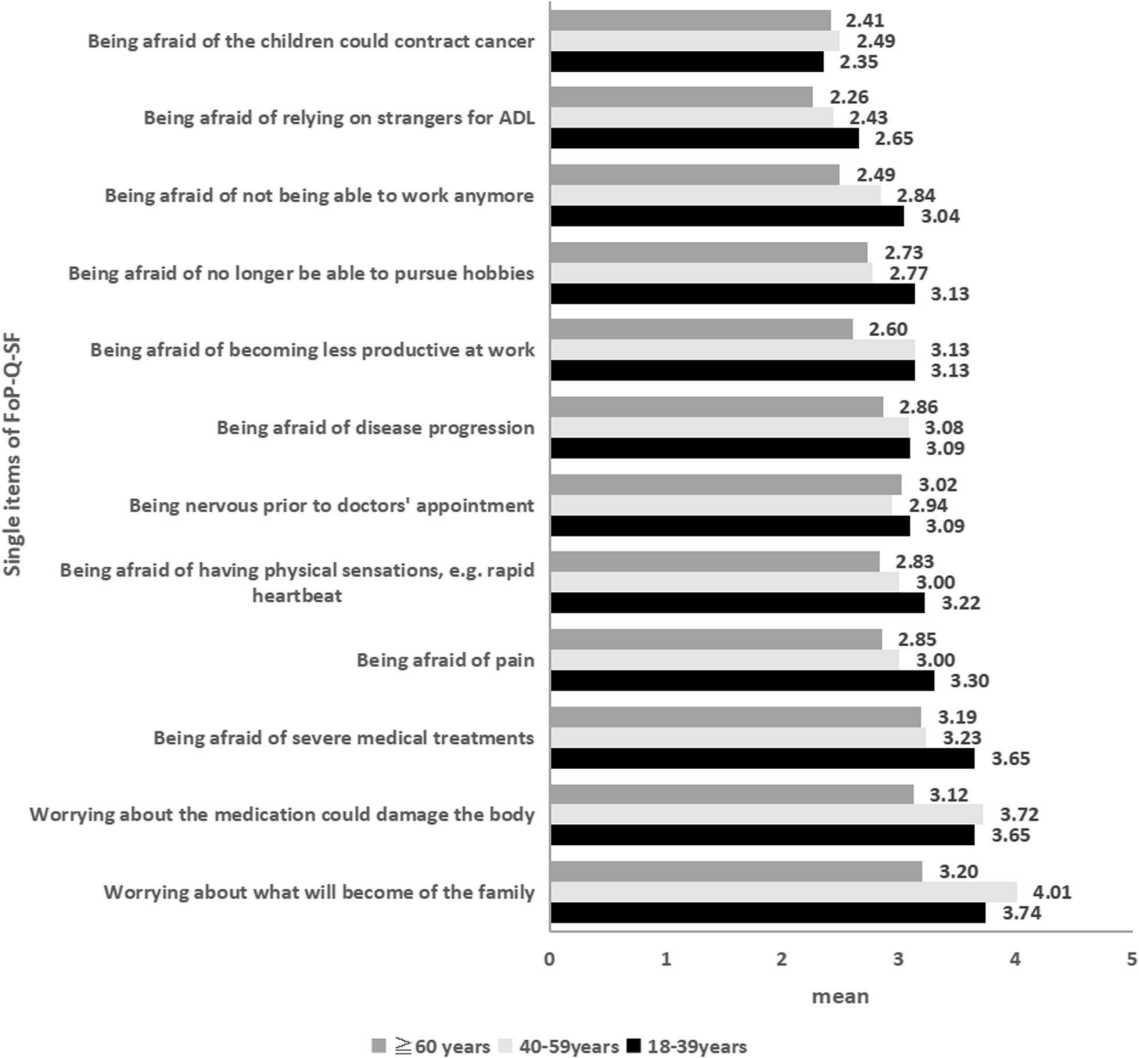
The single-item analysis of FoP between different age groups

We found the mean score of FoP was 35.39 ± 8.03, with scores ranging between 20 and 60. Of the population, 106 (56.4%) patients with scores ≥ 34 had a clinically dysfunctional level of FoP. The single-item analysis (Fig. 1) showed that the top three most frequent fears included worries about what will become of the family, medications that could harm their body, and fear of extensive medical treatments. Notably, these items differed between age cohorts. Patients aged 40–59 years showed significantly higher fears of family-related concerns (*P* < 0.001), potential medication harms (*P* = 0.001). Patients aged 18–39 years and 40–59 years showed significantly higher fears of work-related concerns (*P* = 0.012). We also found that within the subgroup of married/unmarried/separated/widowed patients, the item “worries about what will become of the family” in the single-item analysis was most frequent. The mean score of the item was 3.61 ± 1.10. The patients unmarried 4.33 ± 0.82 and separated 4.00 ± 0.00 showed higher scores of worrying about what will become of the family compared with the married 3.57 ± 1.11 and widowed 3.75 ± 1.26 patients, although the difference was not statistically significant.

### Factors associated with FoP

Factors associated with FoP on univariable analysis are detailed in Table 2. It revealed that high FoP was associated with patient’s age (*P* = 0.004), the time from surgery (*P* < 0.001) and SSRS score (*P* = 0.024). Patients who were younger, the time from surgery (4–6 month), low social support tended to report higher FoP than the older, the time from surgery (0–3 month) and those who had high social support. The diagnosis of collinearity (Table 3) showed that the VIF values of all variables are less than 10, indicating that there is no collinearity between variables. The Pearman correlation analyses showed significant positive correlations between FoP and BIPQ (*r* = 0.199, *P* = 0.006). No correlations were found between FoP and SSRS (*r* = -0.134, *P* = 0.067) as well as SSCQ (*r* = 0.013, *P* = 0.855). Multiple logistic regression analyses showed that age, the time from surgery, and SSRS score were independently associated with higher FoP (Table 4).

**Table 2:** The univariate analysis for FoP (N = 188)

Variable	FoP				
	Total (N = 188) N (%) mean (SD)	Low (N = 82) N (%) mean (SD)	High (N = 106) N (%) mean (SD)	χ^2^/t	*P*-value
Age				11.212	0.004
15–39 years	23 (12.2)	5 (6.1)	18 (17.0)		
40–59 years	79 (42)	29 (35.4)	50 (47.2)		
≧60 years	86 (45.7)	48 (58.5)	38 (35.8)		
Gender				1.265	0.261
Male	99 (52.7)	47 (57.3)	52 (49.1)		
Female	89 (47.3)	35 (42.7)	54 (50.9)		
Educational level				1.725	0.631
Primary school or below	47 (25)	18 (22)	29 (27.4)		
Junior high school	49 (26.1)	20 (24.4)	29 (27.4)		
High school and technical secondary school	43 (22.9)	22 (26.8)	21 (19.8)		
College or above	49 (26.1)	22 (26.8)	27 (25.5)		
Marital status				~	0.228
Unmarried	175 (93.1)	79 (96.3)	96 (90.6)		
Separated	6 (3.2)	1 (1.2)	5 (4.7)		
Widowed	3 (1.6)	0 (0)	3 (2.8)		
Married	4 (2.1)	2 (2.4)	2 (1.9)		
Residence				1.703	0.192
Rural	95 (50.5)	37 (45.1)	58 (54.7)		
Town and city	93 (49.5)	45 (54.9)	48 (45.3)		
Employment				0.134	0.714
No	106 (56.4)	45 (54.9)	61 (57.5)		
Yes	82 (43.6)	37 (45.1)	45 (42.5)		
Family per capita income (monthly) RMB				3.711	0.294
Less than 1000	43 (22.9)	14 (17.1)	29 (27.4)		
1001–3000	64 (34.0)	32 (39.0)	32 (30.2)		
3001–5000	44 (23.4)	18 (22.0)	26 (24.5)		
More than 5000	37 (19.7)	18 (22.0)	19 (17.9)		
Religious beliefs				0.542	0.462
Yes	8 (4.3)	5 (6.1)	3 (2.8)		
No	180 (95.7)	77 (93.9)	103 (97.2)		
Chronic diseases				2.424	0.119
Yes	73 (38.8)	37 (45.1)	36 (34)		
No	115 (61.2)	45 (54.9)	70 (66)		
The time from surgery				19.987	< 0.001
0–3 months	128 (68.1)	70 (85.4)	58 (54.7)		
4–6 months	60 (31.9)	12 (14.6)	48 (45.3)		
Stage of disease at initial diagnosis				0.892	0.345
I	69 (36.7)	27 (32.9)	42 (39.6)		
II	119 (63.3)	55 (67.1)	64 (60.4)		
Chemotherapy/radiotherapy after surgery				1.214	0.271
Yes	56 (29.8)	21 (25.6)	35 (33.0)		
No	132 (70.2)	61 (74.4)	71 (67.0)		
SSRS score	43.79 ± 6.78	45.05 ± 6.72	42.81 ± 6.69	2.269^a^	0.024
SCSQ score	52.05 ± 11.03	53.00 ± 9.72	51.32 ± 11.94	1.035^a^	0.302
BIPQ score	55.74 ± 14.79	54.02 ± 14.35	57.07 ± 15.05	− 1.402^a^	0.163

*SD* standard deviation, *a* t-test, *FoP* Fear of Progression, *SSRS* Social Support Rating Scale, *SCSQ* Simplified Coping Style Questionnaire, *BIPQ* Brief Illness Perception Questionnaire

**Table 3:** Multiple collinearity diagnosis of variables

Variables	Collinearity statistics	
	Tolerance	VIF
Social support	0.754	1.327
Simplified coping	0.814	1.228
Brief illness perception	0.900	1.112
Age	0.614	1.629
Chronic diseases	0.784	1.275
Chemotherapy/radiotherapy after surgery	0.908	1.101
Educational level	0.723	1.383
Marital status	0.921	1.086
Employment	0.745	1.343
The time from surgery	0.877	1.140
Stage of disease at initial diagnosis	0.829	1.206

**Table 4:** Multivariate logistic regression analysis of predictors for FoP (N = 188)

Variables	β	SE	Wald	*P*	OR	95% CI for OR	
						Lower	Upper
Constant	2.292	2.027	1.278	0.258	9.893	–	–
Age(Ref = 15 ~ 39 years)	0.000	–	–	–	1.000	–	–
Age(40 ~ 59 years)	− 1.357	0.658	4.248	0.039	0.257	0.071	0.936
Age(≧60 years)	− 2.245	0.720	9.724	0.002	0.106	0.026	0.434
Chronic diseases	0.201	0.386	0.271	0.603	1.223	0.574	2.606
Chemotherapy/radiotherapy after surgery	0.174	0.393	0.196	0.658	1.190	0.551	2.570
Educational level	− 0.170	0.178	0.913	0.339	0.844	0.595	1.196
Marital status	0.144	0.363	0.157	0.692	1.155	0.567	2.351
Employment	0.332	0.423	0.615	0.433	1.393	0.608	3.190
The time from surgery	1.596	0.412	14.977	< 0.001	4.933	2.198	11.068
Stage of disease at initial diagnosis	− 0.139	0.390	0.126	0.722	0.870	0.405	1.871
SSRS score	− 0.070	0.029	5.713	0.017	0.932	0.880	0.987
SCSQ score	− 0.005	0.017	0.071	0.790	0.995	0.963	1.029
BIPQ score	0.012	0.012	0.961	0.327	1.012	0.988	1.037

Likelihood chi-square = 46.493, *P* < 0.05

## Discussion

The study sought to describe the prevalence and factors associated with FoP in newly diagnosed lung cancer patients to obtain insights on improving the psychological condition of FoP. We found the participants in our sample population experienced high levels of FoP. The identified factors related to FoP among newly diagnosed lung cancer patients were age, the time from surgery, and social support.

In this study, the mean FoP scores are 35.39 ± 8.03. Nearly 56.4% of the participants obtained a high FoP score at a clinically dysfunctional level. A similar study using the same assessment tool revealed that around 36% of young Chinese cancer patients reported high FoP [11]. In comparison, it is lower than the study conducted recently in Jiangxi, China, which reported the scores of FoP are 40.27 ± 7.80, and the prevalence was 77.93% in lung cancer [21]. The time of cancer diagnosis in our sample might contribute to the differences in results. Newly diagnosed lung cancer patients were recruited in our study, but their psychological distress and self-reported experiences are often underreported and inadequately addressed [34]. However, the time of cancer diagnosis may be a key period for physical and emotional transition, and the degree of FoP may increase over time [35]. This may likely be similar to the consideration of the importance of factors producing symptoms consistent with post-traumatic stress disorder (PTSD) [36]. Therefore, more attention should be taken, and more supportive interventions are needed.

We identified a significant difference (*P* = 0.004) in FoP between the 18–39 years, 40–59 years and ≧60 years age groups, with the former reporting higher levels of FoP. The items “Worrying about what will become of the family,” “Worrying about the medications' potential harm to the body,” and “Being afraid of extensive medical treatments” were the top three ranked scores of FoP in this study. In contrast, the items with the three lowest scores were "Being afraid of relying on strangers for ADL,” “Being afraid that the children could contract cancer,” and “Being afraid of not being able to work anymore,” which are consistent with the results of Niu et al. [37]. But not consistent with the study of Götze et al. [38]. Large proportions of patients in our sample are < 60 years old, and most of them are employed. Thus, work-related concerns, including fear of being less productive at work or not being able to work anymore, are likely to appear more important in our sample. Thus, compared with elderly patients, young and middle-aged patients are the primary source of economic income to sustain their families. When diagnosed with cancer, they may face more challenges and suffer from more psychological disorders. In addition, faced with a series of postoperative feelings such as pain, scars and complications, they may be at a higher risk of collapse and distress, and they may be more worried about postoperative prognosis, survival time and impact on their work and family. These findings suggest that the young and middle-aged patients should be followed-up after an operation. Likewise, medical professionals should encourage them to express their self-emotion and provide psychological counseling through evaluation, communication, and empathy [39]. In addition, information and available resources regarding treatment, diet, exercise, and employment protections might be beneficial for reducing their concerns about work and family [40].

Previous studies contented that gender, education level, monthly income, treatment with chemotherapy or radiotherapy, and unemployment were significantly correlated with the FoP [20, 25, 41], which is inconsistent with our research. One possible reason for these findings is the samples and the cultural differences. It may also be associated with the types of cancer, adjuvant treatment, or other factors such as economic status. This study showed that age was the independent predictor of FoP, which is consistent with the research of Nahm [42].This study found that prevalence of FoP was significantly higher among 18–39 years survivors (78.3%) than those diagnosed at 40–59 years (63.29%) and more than 60 years (44.2%). One possible reason is that younger patients may think their illness is unexpectedly, and experience more psychological distress [20].

Consistent with Niu’s [37] study, we found that social support was negatively correlated with FoP in patients with lung cancer after surgery. Social support refers to a certain social network supported by various resources (such as money, emotion and friendship), to help disadvantaged groups in society for free [43]. In particular, offering social support, paying more attention to their personality characteristics such as their favorite social activities, living hobbies and living habits, and focusing on their values from the residents or the spouse, makes patients feel safe, cared for, and respected. Moreover, the internal and external resources that provided for patients can reduce their stress level and promote their optimism in facing diseases, which may improve their psychological health to some extent. Thus, it is imperative for medical professionals to evaluate patient social support and unmet needs and provide them with available interventions to improve their emotional stability [44].

This study showed time from surgery was the independent predictor of FoP. The patients after operation within 3 months experience lower FoP than those after operation in 4–6 months. The analysis may be related to the lack of knowledge and awareness about postoperative complications and postoperative rehabilitation. Patients with lung cancer may be more sensitive, and they pay much attention to postoperative symptoms such as pain, chest tightness, and irritating cough, thus aggravating their psychological burden. The patients after operation in 4–6 months have more time to ruminate about FoP and fears of other symptoms. It is suggested that medical professionals can reconstruct patients' cognition of illness through cognitive behavioral therapy and health education [45] by teaching patients the methods of symptom identification and symptom management after an operation.

### Limitations

This study has several limitations. First, the cross-sectional design may have some biases, such as sample selection bias and information bias, since a small sample from one hospital and the collected self-reported data might affect the results. A further multicenter large-sample investigation with validated instruments is warranted. Second, several potential risk variables, such as anxiety, depression, and quality of life, were not investigated, which should also be considered in future investigations. Third, our study may be limited by not identifying any causal association between tested variables and FoP. A longitudinal study with dyadic analysis is recommended. Finally, our study had only included patients at the early stages of lung cancer (i.e., stage I and II), our single-center and localized findings may not be generalized to patients who are diagnosed with middle to late-stage lung cancer in China.

### Clinical implications

Our findings of this study have many valuable clinical and research implications. Given the high FoP scores of newly diagnosed lung cancer patients, medical professionals should pay more attention and make a more significant effort to help the younger patients who are at risk for clinical FoP coping with the psychological problem. The interventions including cognitive behavior therapy [46], mindfulness-based cognitive therapy [47], acceptance and commitment therapy [48], and problem-solving therapy [49] are recommended. As general considerations, internet and professional guidance can be combined to give patients personalized support in the forms of short films, animations, pictures, face-to-face consultation, and telephone consultation. Moreover, future studies focusing on the cognitive restructuring project that integrates Chinese culture, guidance on how to identify the possible symptoms after lung cancer surgery, and psychological distress of recurrence may be helpful to provide professional psychoeducation and support.

## Conclusions

The study adds to prior work by identifying the prevalence and factors associated with FoP in newly diagnosed lung cancer patients and suggesting suitable strategies for improving patients’ psychological conditions. Our study showed that high FoP is a frequently reported problem among the newly diagnosed lung cancer population, especially those 18–39 years old. The patient’s age, the time from surgery, and social support significantly influenced patient FoP. Overall, the results gleaned from this new investigation is more informative and clinically relevant, and will enable clinicians to manage and treat younger patients who are at risk for clinical FoP. These findings indicate that patients with newly diagnosed lung cancer with high FoP may benefit from professional psychoeducation, psychological interventions, and personalized support.

## Data Availability

The data that support the findings of this study are available from the corresponding author upon reasonable request.

## Abbreviations

FCR: Fear of Cancer Recurrence
FoP: Fear of Progression
SRMI: The Self-Regulation Model of Illness
SCPM: Social-Cognitive Processing Model
IP: Illness Perception
SSRS: Social Support Rating Scale
SCSQ: Simplified Coping Style Questionnaire
BIPQ: Brief Illness Perception Questionnaire
